# Pickled vegetables and the risk of oesophageal cancer: a meta-analysis

**DOI:** 10.1038/sj.bjc.6605372

**Published:** 2009-10-27

**Authors:** F Islami, J-S Ren, P R Taylor, F Kamangar

**Affiliations:** 1 Digestive Disease Research Center, Shariati Hospital, Tehran University of Medical Sciences, Tehran 14117, Iran; 2 International Agency for Research on Cancer, Lyon 69008, France; 3 King's College London, Thames Cancer Registry, London SE1 3QD, UK; 4 Division of Cancer Epidemiology and Genetics, National Cancer Institute, National Institutes of Health, Bethesda, MD 20892-7232, USA; 5 Department of Public Health Analysis, School of Community Health and Policy, Morgan State University, Baltimore, MD 21251, USA

**Keywords:** pickled vegetable, oesophageal cancer, case–control, cohort, meta-analysis

## Abstract

**Background:**

Ecological and experimental studies have suggested a relationship between Asian pickled vegetable consumption and oesophageal squamous cell carcinoma (OSCC), but the results of epidemiological studies investigating the association have been inconsistent. We conducted a meta-analysis of observational studies of this association to evaluate the existing evidence.

**Methods:**

We searched the PubMed, ISI-Web of Science, J-EAST, IndMed, Vip Chinese Periodical, and China National Knowledge Infrastructure databases for all studies published in English or Chinese languages. Pooled results for all studies combined and for several study subgroups were computed.

**Results:**

A total of 34 studies were included in this analysis. The overall random effects odds ratio (OR) and 95% confidence interval (CI) for pickled vegetable consumption was 2.08 (1.66–2.60), but the results were heterogeneous across studies. After excluding the three most influential studies, the respective numbers were 2.32 (1.92–2.81). Similar to the overall association, the majority of subgroup analyses showed a statistically significant association between consuming pickled vegetables and OSCC risk. There were only three prospective studies.

**Conclusion:**

Our results suggest a potential two-fold increased risk of oesophageal cancer associated with the intake of pickled vegetables. However, because the majority of data was from retrospective studies and there was a high heterogeneity in the results, further well-designed prospective studies are warranted.

Eating pickled vegetables has long been suspected as a cause of oesophageal squamous cell carcinoma (OSCC). Cancer registries established in the 1960s showed extremely high incidence rates of OSCC in certain areas of China, such as Linxian (The Coordinating Group for Research on Etiology of Esophageal Cancer in North China, 1975; Tran *et al*, 2005). Known causes of OSCC, most notably tobacco and alcohol consumption could not explain this high incidence (Kamangar *et al*, 2009), so other suggested causes (Yang, 1980), particularly eating pickled vegetables, were investigated.

In high-risk areas of China, pickled vegetables were eaten daily for 9–12 months a year and were an integral part of the diet in some families (Yang, 1980), traditionally prepared by keeping tightly packed moist vegetables in a jar packing for a few weeks or months, allowing fermentation and growth of fungi and yeasts (Yang, 1980; IARC Working Group, 1993) that can generate potentially carcinogenic *N*-nitroso compounds and mycotoxins (Yang, 1980; Cheng *et al*, 1981; Zhang *et al*, 1983). Mutagenicity and carcinogenicity of pickled vegetables have been shown in some experimental and *in vitro* studies (Cheng *et al*, 1980; Yang, 1980; Lu *et al*, 1981).

Although a higher risk of OSCC has been found in areas that used more pickled vegetables (Yang, 1980), case–control and cohort studies have not consistently supported a causal role. IARC Working Group (1993) concluded that there was limited evidence in humans and inadequate evidence in experimental animals for carcinogenicity of pickled vegetables. Since then, many studies have investigated the question, and in this review and meta-analysis we examine the studies of the association of pickled vegetable consumption OSCC risk.

## Materials and methods

We conducted a comprehensive search of the PubMed, ISI-Web of Science, Japanese Science, Technology and Medical Literature (J-EAST), Indian Medlars Center (IndMed), Vip Chinese Periodical Database, and China National Knowledge Infrastructure databases for studies published in English or Chinese languages on oesophageal cancer risk in relation to pickled vegetables consumption upto 10 April 2009. The following terms were used to search text words in the PubMed and the ISI-Web of Science databases: (oesophageal OR oesophageal OR oesophagus OR oesophagus) AND (cancer OR carcinoma OR adenocarcinoma OR neoplasm OR neoplasia OR neoplastic) AND (pickle OR pickled OR moldy OR fermented). Combinations of the above terms were used to search keywords in the J-EAST database. For IndMed database, the first two phrases of the above terms were used. Combinations of the following terms were used for the Chinese databases: 
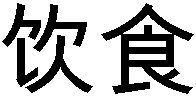
 (diet), 
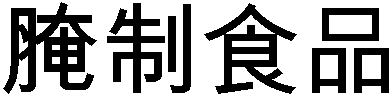
 (pickled food), 
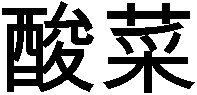
 (Suan Cai, a kind of pickled vegetable), 
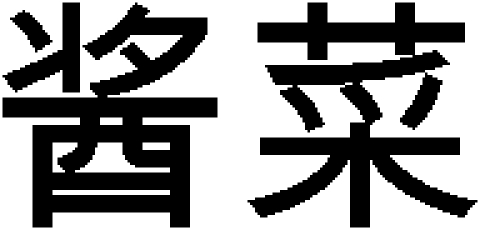
 (salted vegetable), 
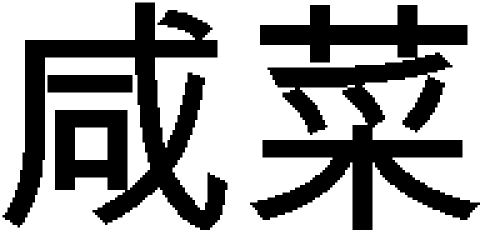
 (salted vegetable), 
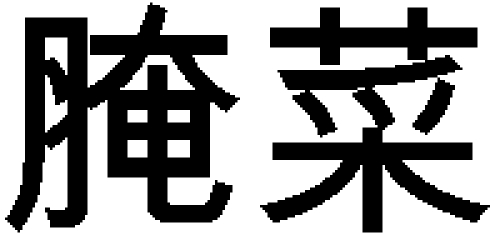
 (pickled vegetable), 
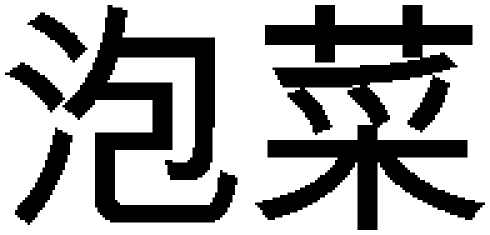
 (pickled vegetable), 
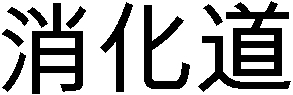
 (digestive tract), 
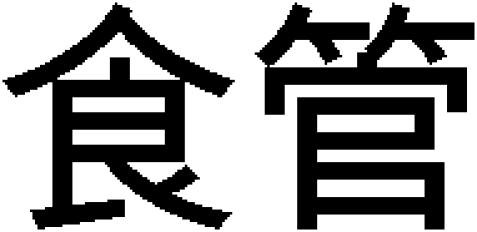
 (oesophagus or oesophageal), 
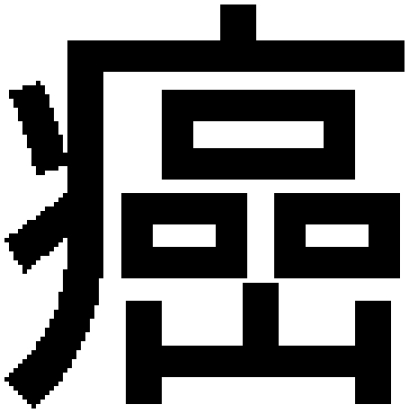
 (cancer or carcinoma), and 
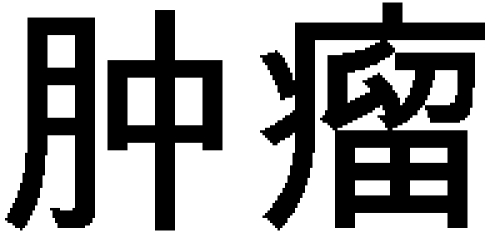
 (tumour). At least two of the authors (FI, JR, or FK) reviewed the publications in English; those in Chinese were reviewed by one author (JR).

We read the abstracts of the retrieved articles; in case of any doubt, we also reviewed the full texts of those articles. Furthermore, references cited in all relevant original and review articles were searched manually. We included only studies with case–control or cohort design, the former limited to those that used control subjects without upper gastrointestinal cancers. Using these approaches, we found 55 full-text articles, among which 2 (Wang *et al*, 1992; Chen *et al*, 2003) reported the results from 3 and 2 individual studies, respectively, making the total number retrieved 58, of these 24 were excluded because: (i) no risk estimates or crude numbers of cases and controls for the exposure-of-interest were reported (Shen *et al*, 1999; Wang *et al*, 2006); (ii) the method of pickling vegetables differed from those in East and Southeast Asia (Onuk *et al*, 2002; Sapkota *et al*, 2008); (iii) results were only reported in combination with other foods (Hou *et al*, 1999; Lu *et al*, 2000; Wang *et al*, 2004; Xie *et al*, 2005; Pan *et al*, 2006) or in combination with other cancers (Li *et al*, 1989; Yu *et al*, 1993; Tong *et al*, 2001; Zhang *et al*, 2001; one of the three studies reported in Chen *et al*, 2003); and (iv) results were also reported in other publications (Chu and Ye, 1992; Chu *et al*, 1992a, 1992b; Guo *et al*, 1994; Cheng *et al*, 1995; Li *et al*, 2001; Xu *et al*, 2002; Li *et al*, 2007; Feng *et al*, 2008a; another one of the three studies reported in Chen *et al*, 2003). Finally, 34 studies remained in the meta-analysis. Although many studies reported the results for oesophageal cancer without specifying any histological subtype, as OSCC constitutes the large majority in China and other places where such pickled vegetables are highly consumed (Kamangar *et al*, 2006, 2009; Eslick, 2009), our findings mainly relate to OSCC.

### Statistical analysis

Wherever available, we extracted details of author, place and year of publication, the actual number of pickled vegetable users as well as non-users by case status, the crude and adjusted odds ratios (OR) and 95% confidence intervals (CI), study design, and the method of selecting controls.

Owing to high heterogeneity across studies, we used random-effects models (DerSimonian–Laird method) to calculate OR and 95% CI for all studies combined. When an individual study presented both crude and adjusted ORs and 95% CIs, we used the maximally adjusted results. If results on multiple consumption categories were given, we collapsed the categories into a single measure of association using fixed-effects models. Depending on the available data, the reference groups included subjects with no or the lowest amount of pickled vegetable consumption. To reduce the influence of outlier studies, we used the command ‘metainf’ (in STATA software, version 10.0) to identify the three most dominant studies in fixed-effects estimates and repeated the analyses without those studies.

We analysed cohort (including nested case–control) separately from case–control studies. As in our previous meta-analyses (Gorouhi *et al*, 2008; Islami and Kamangar, 2008), we present the results for larger studies, defined as those with s.e. ⩽0.5 separately. Subgroup analyses were also carried out on studies with population-based controls. As age is a strong risk factor for OSCC, studies using age-matched controls (group- or individual-matched) were examined separately. As the association with pickled vegetables could be confounded by other factors, for example socio-economic status, we examined studies with adjusted ORs (95% CIs) separately, excluding those with adjustments only for age and sex. We also calculated summary OR (95% CI) for the subgroup that reported the numbers or proportions of cases and controls who consumed pickled vegetables, because we were more certain that the calculation of OR (95% CI) was correct in these studies.

Studies from Linxian, a high-risk area in China, showed very heterogeneous results, consumption among controls ranged from 0 to approximately 60%, and point-estimates of association varied widely on both sides of null. The observed heterogeneity may be because of the mass public health campaigns in the 1970s in Linxian and some other regions, which advised people to avoid consumption of pickled vegetables to reduce the risk of oesophageal cancer (Sakaguchi *et al*, 2005; Tran *et al*, 2005). In consequence, some subjects may have reduced their intake of pickled vegetables or under-reported their use in the survey. We therefore, compared studies from Linxian *vs* all others, and from Mainland China *vs* all others (including Hong Kong, Taiwan, Japan, and India). As these public health campaigns subsided in the 1990s, we also carried out subgroup analyses for earlier studies (initiated in 1990 or earlier) and more recent studies (initiated after 1990). We investigated whether there was any difference in the risk estimates between English and Chinese papers.

We used Begg's funnel plot to examine small study effects (Sterne *et al*, 2001), Begg and Mazudmar's method (Begg and Mazumdar, 1994) to calculate *P*-value for rank correlation and Egger's weighted regression method (Egger *et al*, 1997) to calculate *P*-value for publication bias. To examine heterogeneity, the *Q*-statistic (using Mantel–Haenszel weights) and the *I*^*2*^ statistic were calculated (Higgins *et al*, 2003). Throughout the paper, two-sided *P*-values <0.05 were considered as statistically significant.

## Results

Summary characteristics of the 34 included studies are presented in Table 1; more detailed information is given in Supplementary Table 1. There were 3 prospective and 31 case–control studies; approximately half of the studies were population based, the other half clinic based. Most studies (*n*=29) were from Mainland China; 56% (*n*=19) were in Chinese.

**Figure 1:**
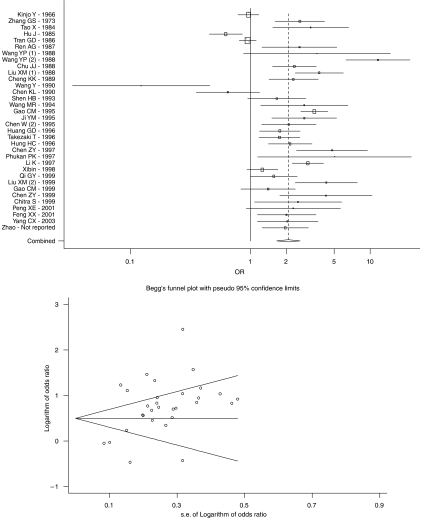
Forest plot and Begg's funnel for the association between consumption of pickled vegetables and oesophageal cancer. Studies are sorted in order of starting year.

For the overall association, the OR (95% CI) was 2.08 (1.66–2.60). The *P*-value associated with the *Q*-statistic was <0.001, and the *I*^2^-statistic was 88%, suggesting very high heterogeneity (Table 2). A forest plot and Begg's funnel plot for overall associations are shown in Figure 1, wherein the distribution of dots appeared to be non-symmetric, and *P*-value for bias in Egger's weighted regression method was 0.01, suggesting publication bias. However, there was no evidence for bias using Begg and Mazumdar's method (*P*-value for rank correlation=0.30). After excluding the three studies with the highest influence on overall results (Hu *et al*, 1994; Kinjo *et al*, 1998; Tran *et al*, 2005), the OR (95% CI) changed to 2.32 (1.92–2.81).

Table 2 shows the results of subgroup analyses, the majority of which showed a significant association between consumption of pickled vegetables and oesophageal cancer risk. Results from prospective studies (*n*=3) were insignificant (OR=1.52; 95% CI 0.82–1.63), whereas case–control studies showed highly significant results (OR=2.18; 95% CI 1.75–2.73). After excluding small studies, defined as those having a s.e. >0.5, the summary OR (95% CI) was 2.09 (1.67–2.63). Summary ORs (95% CIs) for studies with population-based controls, age-matched controls, and adjusted results were comparable to those of large studies (Table 2). Studies that reported actual data on pickled vegetable consumption among cases and controls showed OR (95% CI) of 2.55 (2.01–3.24).

Studies from Linxian (*n*=4), showed a marginally significant association (OR=1.51; 95% CI 0.98–2.33), whereas the summary OR (95% CI) for those conducted elsewhere was 2.17 (1.70–2.78). Studies initiated in 1990 or earlier showed a moderate association (OR=1.73; 95% CI 1.14–2.62), while those initiated after 1990 showed a more than two-fold increased risk (OR=2.28; 95% CI 1.89–2.76). The summary ORs (95% CI) for studies from China (Mainland) and elsewhere were 2.10 (1.63–2.69) and 1.96 (1.12–3.43), respectively. The summary ORs (95% CIs) for studies retrieved from English and Chinese literature were 2.10 (1.47–3.00) and 2.10 (1.65–2.67), respectively.

## Discussion

We found an approximately two-fold increased risk of OSCC associated with pickled vegetable use in the overall analysis and in the majority of subgroup analyses. However, this association was insignificant in prospective studies or in studies from Linxian. There was also very high heterogeneity in the strength of associations between studies.

None of our multiple subgroup analyses substantially reduced heterogeneity, the *I*^2^-index for heterogeneity remaining above 60%. However, case–control studies, those with age-matched controls, studies that reported the prevalence of use in cases and controls, studies outside Linxian, and studies conducted after 1990, when public health campaigns against pickled vegetable use had subsided, all showed stronger associations.

Two of the three cohort studies showed no increased risk. The results of dietary cohort studies are usually more reliable than retrospective case–control studies, because the results of the former are not subject to recall bias or interviewer bias. However, in this analysis, there are reasons to dispute the summary results from the cohort studies. One of these studies (Tran *et al*, 2005) with a large sample size was from Linxian in a time when strong recommendations had been made against pickled vegetable use. Such use in that study was very low (close to zero), which may be due to residents having temporarily discontinued using this food item, or may have felt uncomfortable about reporting their consumption. In either case, the results would be flawed due to exposure misclassification.

After excluding the three most influential studies (Hu *et al*, 1994; Kinjo *et al*, 1998, 2003; Tran *et al*, 2005) the results remained highly heterogeneous. One of these studies (Tran *et al*, 2005) was conducted in the very high-risk regions of Linxian and, as discussed above, may have been subject to exposure misclassification. We tested for publication bias using two methods. Although the *P*-value from Egger's weighted regression method suggested a publication bias, but that from Begg and Mazumdar's method did not suggest such a bias. The main reason for the low *P*-value from Egger's method may not be publication bias, but rather due to the null results from large studies conducted in Linxian in the 1980s *vs* increased risks found in smaller studies from other places.

Although the above results do not prove an association, they strongly suggest that the use of pickled vegetables increases OSCC risk. This association is more likely if the studies in Linxian, at the time of mass public health campaigns or soon after, were subject to exposure misclassification. On the other hand, the majority of data come from case–control studies, which are subject to recall or interviewer bias. We therefore believe that the association needs further investigations in well-designed prospective studies, with validated questionnaires and trained interviewers.

Extracts of pickled vegetables have shown mutagenic and transforming activities as well as promoting effects *in vitro* (Cheng *et al*, 1981), inducing hyperplasia and dysplasia in oesophageal epithelium and tumours in rodents (Yang, 1980). Heavy infestation of pickled vegetables with fungi is reported from Linxian (Yang, 1980). Some common fungi can reduce nitrate to nitrite and also increase amines content of pickled vegetables (Li *et al*, 1986). This may facilitate the formation of *N*-nitroso compounds, which are strong oesophageal carcinogens in several animal models (Kamangar *et al*, 2009). Roussin red methyl ester, a non-alkylating nitroso compound with promoting effect *in vitro*, has been identified in pickled vegetables samples from Linxian in much higher concentrations than in samples from a low-incidence areas (Cheng *et al*, 1981; Zhang *et al*, 1983). Fumonisin mycotoxins have been shown to cause liver and kidney tumours in rodents (Kamangar *et al*, 2009). Furthermore, a synergistic interaction between mouldy food and *N*-nitroso compounds has been reported (Yang, 1980).

This meta-analysis has several strengths. We covered several databases, including those indexing publications in the areas where pickled vegetables are highly consumed; we searched publications in Chinese as well as in English. The meta-analysis presents results from 34 studies with a large number of cases and controls. As in any meta-analysis of observational studies, however, combining studies conducted in different populations with various qualities of design to obtain summary ORs and 95% CIs may be misleading, and summary statistics needs to be interpreted with caution. Few prospective studies have investigated pickled vegetables use and OSCC, so most data were from case–control studies, in which food intake data may be subject to recall and interviewer bias.

In summary, our results suggest a two-fold higher risk of OSCC associated with the intake of pickled vegetables. However, because most data were from retrospective studies with high heterogeneity in the results, further well-designed prospective studies with validated questionnaires are warranted.

## Figures and Tables

**Table 1:** A summary of studies on the association between pickled vegetables and risk of oesophageal cancer

**Study/Literature**	**Study place (period)**	**Case/control**	**Studied variable**	**Consumption rate/Matched/Adjusted^a^**	**Study design**
Kinjo *et al*, 1998/E	Japan; Kagoshima, Okayama, Hyogo, Osaka, Aichi, Miyagi (1966–1981)	440/220 272	Pickles	No/No/Yes	Cohort; the outcome was oesophageal cancer death
Zhang *et al*, 2000/C	China; Cixian (1973–1997)	350/350	P. V.	Yes/Yes/No	P-B CCS
Tao *et al*, 1999/E	China; Shanghai (1984–1988)	71/1122	P. V.	Yes/No/Yes	Nested CCS (only men); the outcome was oesophageal cancer death
Hu *et al*, 1994/E	China; Heilongjiang (1985–1989)	196/392	P. Chinese cabbage	No/Yes/Yes	H-B CCS
Tran *et al*, 2005/E	China; Linxian (1986–2001)	1958/29 584	P. V.	No/No/Yes	Cohort
Ren and Han, 1991/C	China; Tangshan (1987–1989)	112/112	P. V.	Yes/Yes/Yes	H-B CCS
Wang *et al*, 1992 (Study I)/E	China; Shanxi: high-risk area (1988–1989)	210/396	P. V. juice	Yes/Yes/Yes	CCS; H-B cases, P-B controls
Wang *et al*, 1992 (Study II)/E	China; Shanxi: moderate-risk area (1988–1989)	116/396	P. V. juice	Yes/Yes/Yes	CCS; H-B cases, P-B controls
Chu *et al*, 1993/C	China; Huaian (1988–1989)	151/151	P. V.	Yes/Yes/No^b^	P-B CCS
Liu *et al*, 2001a/C	China; Tianjin (1988–1989)	165/165	P. V.	Yes/Yes/No	P-B CCS (only men)
Cheng *et al*, 1992/E	Hong Kong (1989–1990)	400/1598	P. V.	Yes/Yes/Yes	H-B CCS
Wang *et al*, 1993/C	China; Yanting (1990–1991)	155/155	P. V.	No/Yes/Yes	H-B CCS
Chen *et al*, 1995/C	China; Jintang (1990–1992)	148/296	P. V.	Yes/Yes/No	P-B CCS
Shen *et al*, 1997/C	China; Huaian (1993–1994)	158/158	P. V.	Yes/Yes/No^b^	P-B CCS
Wang *et al*, 1999/C	China; Yangzhong (1994–1995)	68/68	P. V.	No/Yes/Yes	H-B CCS
Gao *et al*, 1999/E	China; Yangzhong (1995)	81/234	P. V.	Yes/Yes/Yes	P-B CCS
Ji, 1999/C	China; Linxian (1995–1996)	67/65	P. V.	Yes/No/Yes	H-B CCS
Chen *et al*, 2003/C	China; Linxian (1995–1997)	702/702	P. V.	No/Yes/Yes	P-B CCS
Huang *et al*, 2000/C	China; Meizhou (1996–1999)	150/150	P. V.	No/Yes/Yes	H-B CCS
Takezaki *et al*, 2001/E	China; Pizhou (1996–2000)	199/333	P. V.	No/No/Yes	CCS; H-B cases, P-B controls
Hung *et al*, 2004/E	Taiwan (1996–2002)	364/532	Preserved and P. V.	Yes/Yes/Yes	H-B CCS (only men)
Chen *et al*, 2000/C	China; Rugao (1997–1998)	100/100	P. V.	No/Yes/No	H-B CCS
Phukan *et al*, 2001/E	India; Assam (1997–1998)	502/1004	Pickles	Yes/Yes/Yes	H-B CCS
Li and Yu, 2003/E	China; Chaoshan (1997–2000)	1248/1248	Pickles	No/Yes/Yes	H-B CCS
Xibib *et al*, 2003/E	China; Linxian (1998–2000)	211/633	Salted or P. V.	No/Yes/Yes	P-B CCS
Qi *et al*, 2001/C	China; Pizhou (1999)	103/103	P. V.	No/Yes/Yes	P-B CCS
Liu *et al*, 2001b/C	China; Tianjin (1999)	86/158	P. V.	No/No/Yes	P-B CCS
Gao *et al*, 2001/C	China; Huai’an (1999–2000)	93/98	P. V.	No/Yes/Yes	H-B CCS
Chen *et al*, 2004/C	China; Rugao (1999–2000)	100/100	P. V.	No/Yes/Yes	H-B CCS
Chitra *et al*, 2004/E	India; Coimbatore (1999–2000)	90/90	Pickles	Yes/Yes/No	H-B CCS
Peng *et al*, 2005/C	China; Anxi (2001–2002)	237/237	P. V.	No/Yes/Yes	H-B CCS
Feng *et al*, 2008b/C	China; Changzhi (2001–2005)	201/201	P. V.	Yes/Yes/No	H-B CCS
Yang *et al*, 2005/E	China; Yanting (2003–2004)	185/185	P. V.	Yes/Yes/Yes	CCS; H-B cases, P-B controls
Zhao *et al*, 2003/C	China; Feicheng (not reported)	217/212	P. V.	Yes/No/Yes	CCS. Origin of cases was not reported. Controls were healthy residents in a high-risk village

Abbreviations: C=Chinese; CCS=case–control study; E=English; H-B=hospital-based; P=pickled; P-B=population-based; V=vegetable.

a This column shows whether the proportion of cases and controls who consumed the studied variables were reported/group- or individual-matching of controls to cases for age/reporting adjusted ORs for the association between consumption of pickled vegetables and cancer.

b Adjusted ORs were also reported for these studies but because 95% CIs were not presented (Supplementary Table 1), we included their univariate results in the meta-analysis.

**Table 2:** Summary statistics for the association between consumption of pickled vegetables and oesophageal cancer

	**Number of studies**	***Q*-statistic^a^**	***P*-value^b^**	***I*^2^ (%)^c^**	**Random effects OR (95% CI)**
All studies	34	282.18	<0.001	88	2.08 (1.66–2.60)
After excluding the three most influential studies	31	120.45	<0.001	75	2.32 (1.92–2.81)
Prospective studies	3	10.26	0.006	81	1.52 (0.82–1.63)
Case-control studies	31	179.25	<0.001	83	2.18 (1.75–2.73)
Large studies (standard error ⩽0.5)	30	259.42	<0.001	89	2.09 (1.67–2.63)
Studies with population-based controls	18	185.63	<0.001	91	2.11 (1.56–2.85)
Studies with age-matched controls	27	171.69	<0.001	85	2.14 (1.66–2.76)
Adjusted results	24	196.16	<0.001	88	2.15 (1.64–2.81)
Studies reporting consumption rates among cases and controls	18	57.61	<0.001	70	2.55 (2.01–3.24)
Studies from Linxian, China	4	18.69	<0.001	84	1.51 (0.98–2.33)
Studies not from Linxian, China	30	220.89	<0.001	87	2.17 (1.70–2.78)
Studies initiated in 1990 or earlier^d^	13	160.43	<0.001	93	1.73 (1.14–2.62)
Studies initiated after 1990^d^	20	51.69	0.002	63	2.28 (1.89–2.76)
Studies from China (Mainland)	29	246.64	<0.001	89	2.10 (1.63–2.69)
Studies not from China (Mainland)	5	25.31	<0.001	84	1.96 (1.12–3.43)
Studies published in English literature	15	198.34	<0.001	93	2.10 (1.47–3.00)
Studies published in Chinese literature	19	60.06	<0.001	70	2.10 (1.65–2.67)

Abbreviations: CI=confidence interval; OR=odds ratio.

a Chi-square *Q*-statistic for homogeneity.

b *P*-value for the *Q*-statistic.

c Higgins *I*^2^ statistic for heterogeneity.

d One study was excluded from these analyses because the starting year was not reported.
