# Supplementary material for: Pickled vegetables and the risk of oesophageal cancer: a meta-analysis
Source: Br J Cancer. 2009 Oct 27;101(9):1641–7. doi: 10.1038/sj.bjc.6605372 (PMC2778505; doi:10.1038/sj.bjc.6605372)
Supplement: Supplementary Table 1 [file 6605372x1.doc]

Supplementary Table 1. Characteristics of studies on the association between pickled vegetables and risk of oesophageal cancer

| **First author; year of publication (Country; period of study)** | **Case / control**  **Study design** | **Studied variables** | **Percentage of cases/controls** | **RR (95% CI)** | **Comments**  **1. histological subtypes of OC; if available, 2. control matching criteria, if applicable; and 3. adjustments for the presented results. When any information other is given, it is presented as “Note”.** |
| --- | --- | --- | --- | --- | --- |
| Kinjo et al, 1998  (Japan; 1966-1981) | 440 / 220272  Cohort; the outcome was OC death. | **Pickles**  Every meal  Once/day  ≤ 1-3 times/w | 64.6/NR  19.3/NR  16.1/NR | 1  0.9 (0.6-1.1)  1.0 (0.7-1.3) | 1. The OC diagnosis method was not reported. No information about subtypes.  2. Controls were non-cancer cohort members; no matching was reported.  3. Adjusted for age, sex, prefecture, occupation, vegetable intake, and tobacco and alcohol use. |
| Zhang et al, 2000  (China;  1973-1997) | 350 / 350  P-B CCS. | **P. Chinese cabbage**  Seldom  Often  **P. V. (other than cabbage)**  Seldom  Often | 94.9/98.3  5.1/1.7  82.0/91.1  18.0/8.9 | 1  3.40 (1.22-9.45)  1  2.59 (1.61-4.16) | 1. 76.0% of cases were diagnosed clinically, 22.3% by imaging techniques, 1.5% during surgery (0.3% unknown). No information about subtypes.  2. Individually matched for age, sex, occupation and residence.  3. Results were not adjusted.  Note: Results for P. V. (other than cabbage) consumption were included in the meta-analysis. |
| Tao et al, 1999  (China; 1984-1988) | 71 / 1122  P-B CCS within a cohort with only male subjects; the outcome was OC death. | **P. V.**  < 3 months/y  ≥ 3 months/y | 62.0/77.6  38.0/22.4 | 1  3.19 (1.54-6.59) | 1. The OC diagnosis method was not reported. No information about subtypes.  2. Controls were the 1% of total non-cancer cohort members, stratified by community. No other matching was reported.  3. Adjusted for age, drinking eater source and mutagenicity, history of disease, tobacco and alcohol use, some dietary patterns, including P. V. intake, some occupational exposures, education, and monthly food expenses. |
| Hu et al, 1994  (China; 1985-1989) | 196 / 392  H-B CCS. | **P. Chinese cabbage**  ≤18.5 kg/y  > 18.5-30.0 kg/y  > 30.0-45.0 kg/y  > 45.0 kg/y | NR/NR (see Study design, comments) | 1  0.7 (0.4-1.2)  0.5 (0.3-0.9)  0.7 (0.4-1.2)  *P* for trend = 0.57 | 1. All cases had histologically confirmed OC. No information about subtypes.  2. Individually matched for age, sex, and area of residence  3. Matched results adjusted for tobacco and alcohol use, income, and occupation.  Note: cutpoints for the categories (quartiles) were according to distribution among controls. |
| Tran et al, 2005  (China; 1986-2001) | 1958 / 29584  Cohort. | **P. V.**  0 times/y  ≥ once/y | NR/100  NR/0 | 1  0.95 (0.81-1.12) | 1. All cases had histologically confirmed OC; only OSCC cases.  2. Controls were the non-cancer cohort members; no matching was reported.  3. Adjusted for age and sex. |
| Ren and Han, 1991  (China; 1987-1989) | 112 / 112  H-B CCS. | **P. V.**  **(each winter and spring)**  Non-user  User  **(frequency)**  <once/2-3 day  once/2-3 day  > once/2-3 day | 49.1/67.0  50.9/33.0  49.1/67.0  41.1/26.8  9.8/6.2 | 1  2.57 (1.25-5.25)  1  2.07  2.01  *P* for trend<0.05 | 1. All cases were diagnosed by X-ray, histology, or surgery; 85.5% were OSCC.  2. Individually matched for age, sex, occupation, and residence area.  3. Adjusted for drinking tea, proportion of flour and rice consumption, and intake of moldy food, egg, and hot food.  Note: the OR for “Consumption in each winter and spring” variable was used in the meta-analysis, because 95% CIs were not presented for the other variable. |
| Wang et al, 1992  (China; 1988-1989) | 210 / 396  CCS; H-B cases, P-B controls. High-risk area. | **P. V.**  **P. V. juice**  Never, rarely  Sometimes, often | NR/NR  96.7/99.0  3.3/1.0 | NS/NR  1  3.6 (1.1-18.4) | 1. All cases were diagnosed by X-ray; 72% were confirmed histologically. No information about subtypes.  2. Controls were frequency matched on age, sex, and residence area.  3. Adjusted for age, sex, and farm/nonfarm occupation.  Note: the OR for “P. V. juice” variable was used in the meta-analysis, because ORs (95% CIs) were not presented for the other variable. |
| Wang et al, 1992  (China; 1988-1989) | 116 / 396  CCS; H-B cases, P-B controls. Moderate-risk area. | **P. V.**  **P. V. juice**  Never, rarely  Sometimes, often | NR/NR  44.0/88.7  56.0/11.3 | NS/NR  1  11.6 (6.3-21.6) | 1. All cases were diagnosed by X-ray; 83% were confirmed histologically. No information about subtypes.  2. Controls were frequency matched on age, sex, and residence area.  3. Adjusted for age, sex, and farm/nonfarm occupation.  Note: the OR for “P. V. juice” variable was used in the meta-analysis, because ORs (95% CIs) were not presented for the other variable. |
| Chu et al, 1993  (China;  1988-1989) | 151 / 151  P-B CCS. | **P. V.**  **(univariate analysis)**  < 15kg/y or duration <15 y  ≥ 15kg/y and duration ≥ 15 y  **(multivariate analysis)**  <15kg/y or duration <15 y  ≥ 15kg+/y and duration ≥ 15 y | 45.0/55.6  55.0/44.4  NR/NR | 1  2.33 (2.03-4.70)  1  3.52 (*P* = 0.0485) | 1. 84% of cases were diagnosed by histology, cytology, X-ray or surgery. No information about subtypes.  2. Individually matched for age, sex, residence area and occupation.  3. Matched results adjusted for cancer family history, drinking pond-ditch water, drinking tea, fresh vegetable and hot food.  Note: Results from the univariate analysis were used in the meta-analysis, because 95% CI was not presented for the multivariate analysis. |
| Liu et al, 2001a  (China; 1988-1989) | 165 / 165  P-B CCS; only men. | **P. V.**  Seldom  Often | 40.0/71.5  60.0/28.5 | 1  3.76(2.37-5.95) | 1. All cases were diagnosed by histology or X-ray. No information about subtypes.  2. Individually matched for age, nationality and education.  3. Matched results were not adjusted. |
| Cheng et al, 1992  (China, Hong Kong; 1989-1990) | 400 / 1598  H-B CCS. | **P. V.**  < once/y  < once/month  1-3 times/month  1-3 times/w  4-6 times/w  Daily or more | 2.8/5.7  6.0/6.1  42.6/52.2  40.1/34.3  5.0/1.4  3.5/0.3 | 1  1.66 (0.06-4.43)  1.51 (0.67-3.39)  2.09 (0.92-4.47)  2.96 (2.03-19.39)  13.12 (2.57-66.93)  *P* for trend < 0.001 | 1. All cases had histologically confirmed OC (85% OSCC, 12% OAC, and 3% other).  2. Individually matched for age and sex.  3. Matched results adjusted for age, education, and birth place. |
| Wang et al, 1993  (China;  1990-1991) | 155 / 155  H-B CCS. | **P. V.**  Seldom  Often | NR/NR | 1  0.12 (0.03-0.46) | 1. Ten cases were diagnosed by balloon cytology, 75 by gastroscopy and biopsy, 64 by X-ray, and 6 by surgery. No information about subtypes.  2. Individually matched for age, sex, nationality, occupation and residence.  3. Matched results adjusted for hot food, drinking non-boiled water, kin, OC family history, moldy food, personal character, little fresh vegetable, alcohol use, coarse food grain, vinegar, meat and cold food. |
| Chen et al, 1995  (China;  1990-1992) | 148 / 296  P-B CCS. | **P. V.**  Seldom  Often | 27.0/26.0  73.0/74.0 | 1  0.65 (0.36-1.21) | 1. All cases were confirmed by imaging techniques, during surgery, or by histology. No information about subtypes.  2. Individually matched for age, sex and residence area.  3. The matched results were not adjusted. |
| Shen et al, 1997  (China ; 1993-1994) | 158 / 158  P-B CCS. | **P. V.**  **(univariate analysis)**  < 5 kg/y  ≥ 5 & < 13 kg/y  ≥ 13 & < 20 kg/y  ≥ 20 kg/y  **(multivariate analysis)**  Variable categories as above | 16.4/22.2  38.0/43.0  32.9/27.2  12.7/7.6  NR/NR | 1  1.31  1.73  2.27  OR=1.31 (*P*=0.02) | 1. All cases were diagnosed by endoscopy or histology. No information about subtypes.  2. Individually matched for age, sex, residence area and occupation.  3. Adjusted for oesophageal cancer family history, drinking pond-ditch water and eating onion or garlic.  Note: Results from the univariate were used in the meta-analysis (95% CIs calculated using raw data), because 95% CI was not presented for the multivariate analysis. |
| Wang et al, 1999  (China; 1994-1995) | 68 / 68  H-B CCS. | **P. V.**  < 2 times/month  ≥ 2 times/month | NR/NR | 1  2.82 (1.22-6.52) | 1. All cases had histologically confirmed OC. No information about subtypes.  2. Individually matched for age and sex.  3. Matched results adjusted for education, alcohol and tobacco use. |
| Gao et al, 1999  (China; 1995) | 81 / 234  P-B CCS. | **P. V.**  Almost never  Occasionally  Frequently | 2.5/8.1  49.3/53.4  48.1/38.5 | 1  3.20 (2.23-4.58)  3.69 (2.53-5.39) | 1. All cases had histologically confirmed OC. No information about subtypes.  2. Individually matched to OC and stomach cancer cases for age, sex, and neighborhood. For the analysis, the enrolled controls for the 2 types of cancer were combined.  3. Adjusted for age and sex. |
| Ji, 1999  (China;  1995-1996) | 67 / 65  H-B CCS. | **P. V.**  **(10-20 ys ago)**  Non-user  User  **(frequency, 10-20 ys ago)**  Nil  1-150 times/y  151-300 times/y  > 300 times/y  **(frequency, in last 10 ys)**  Nil  1-150 times/y  151-300 times/y  > 300 times/y | 22.4/41.5  77.6/58.5  22.4/41.6  28.4/21.5  23.9/21.5  25.3/15.4  50.7/64.6  19.4/12.3  19.4/15.4  10.5/7.7 | 1  1.69 (1.17-2.45)  1  2.44 (0.87-6.96)  2.06 (0.71-6.00)  3.06 (1.00-9.51)  P for trend=0.03  1  2.01 (0.68-6.07)  1.61 (0.57-4.56)  1.73 (0.43-7.52)  *P* for trend=0.19 | 1. All cases had histological confirmed OC. No information about subtypes.  2. No matching was reported.  3. Adjusted for tea consumption, smoking, drinking clean water, intake of salt, fried food, moldy sweet potato, soy food, fruit, indrawn character, oesophageal cancer history of first-level relative.  Note: We included “consumption times/year 10-20 years ago” variable in the meta-analysis, since it could be a more appropriate index, compared to consumption frequency in recent year, considering the potential latency between exposure and development of the cancer. |
| Chen et al, 2003  (China; 1995-1997) | 702 / 702  P-B CCS. | **P. V.**  Seldom  Often | 93.2/NR  6.8/NR | 1  2.1 (1.2-3.4) | 1. No information about OC diagnosis methods or subtypes was reported.  2. Individually matched for age, sex and residence.  3. Adjusted for age and smoking. |
| Huang et al, 2000  (China;  1996-1999) | 150 / 150  H-B CCS. | **P. V.**  Seldom  Often | NR/NR | 1  1.76 (1.19-2.60) | 1. All cases had histologically confirmed OC. No information about subtypes.  2. Individually matched for age, sex and residence area.  3. Matched results adjusted for alcohol use, rapid eating, tomato, sausage, fruit and cancer family history. |
| Takezaki et al, 2001  (China; 1996-2000) | 199 / 333  CCS; H-B cases, P-B controls. | **P. V.**  Almost never  0.5-4.0 kg/y  4.5-10.0 kg/y  > 10.0 kg/y | NR/NR (see Study design, comments) | 1  0.98 (0.48-1.98)  2.16 (1.12-4.18)  2.36 (1.20-4.56)  *P* for trend < 0.001 | 1. All cases had histologically confirmed OC. No information about subtypes.  2. Individually matched for age, sex and ethnicity.  3. Adjusted for age, sex, and tobacco and alcohol use.  Note: according to the article, the percentage of P. V. consumption in whole study area was 40% (results from an ecological study). |
| Hung et al, 2004  (Taiwan; 1996-2002) | 364 / 532  H-B CCS; only men. | **Preserved & P. V.**  **(age 20-40 ys)**  < once/w  ≥ once/w  **(age > 40 ys)**  < once/w  ≥ once/w | 74.6/84.9  25.4/15.1  80.7/83.6  19.3/16.4 | 1  1.9 (1.1-3.4)  1  2.5 (1.3-4.5) | 1. All cases had histologically confirmed OC; only OSCC cases.  2. Individually matched for age and hospitalization date.  3. Adjusted for age, education, ethnicity, source of hospital, and tobacco and alcohol use.  Note: Results for both age groups were mixed together in the meta-analysis. |
| Chen et al, 2000  (China; 1997-1998) | 100 / 100  H-B CCS. | **P. V.**  ≤ once/day  > once/day | NR/NR | 1  4.80 (2.42-9.49) | 1. All cases had histologically confirmed OC; only OSCC cases.  2. Individually matched for age, sex and residence area.  3. The matched results were not adjusted. |
| Phukan et al, 2001  (India; 1997-1998) | 502 / 1004  H-B CCS. | **Pickles**  Never  Occasionally  All meals | 17.1/32.6  68.7/64.1  14.2/3.3 | 1  2.3 (1.1-6.3)  10.4 (4.6-16.9) | 1. All cases had histologically confirmed OC. No information about subtypes.  2. Individually matched for age and sex initially. Some cases and controls were dropped later on; the pairing identity was not retained.  3. Adjusted for education, income, and tobacco and alcohol use. |
| Li and Yu, 2003  (China; 1997-2000) | 1248 / 1248  H-B CCS. | **Pickles**  ≤ once/w  2-3 times/w  > 3 times/w  Daily | NR/NR | 1  2.85 (1.7-4.7)  3.02 (1.8-5.1)  3.22 (1.9-5.5)  *P* for trend < 0.01 | 1. All cases had histologically confirmed OC; only OSCC cases.  2. Individually matched for age, sex, and hospital.  3. Adjusted for age, sex, income, residence, occupation, and tobacco and alcohol use. |
| Xibib et al, 2003  (China; 1998-2000) | 211 / 633  P-B CCS. | **Salted or P. V.**  Nil  <10 g/w  10-39 g/w  ≥ 40 g/w | 62.1 / NR  5.2 / NR  20.4 / NR  12.3 / NR | 1  0.79 (0.29-1.01)  1.97 (0.98-4.01)  1.32 (0.62-2.84)  *P* for trend = 0.17 | 1. All cases had histologically confirmed OC (83% OSCC, 17% OAC).  2. Individually matched for age, sex, and neighborhood.  3. Matched results adjusted for age, income, resident space, education level, intake of bean or bean products, vegetable, vinegar, onion or garlic, and preference for salted food. |
| Qi et al, 2001  (China;  1999) | 103 / 103  P-B CCS. | **P. V.**  Seldom  Often | NR/NR | 1  1.57 (1.00-2.45) | 1. 66% of cases were diagnosed by histology. No information about subtypes.  2. Individually matched for age, sex and residence area.  3. Matched results adjusted for eating speed, food salt degree, and intake of moldy food and garlic. |
| Liu et al, 2001b  (China;  1999) | 86 / 158  P-B CCS. | **P. V.**  <100g/w  ≥ 100g/w | NR/NR | 1  4.30 (1.98-6.50) | 1. All cases had histologically confirmed OC. No information about subtypes.  2. Controls were randomly selected from general population; no matching was reported.  3. Adjusted for drinking tea, and intake of vegetables, fruit and hot food. |
| Gao et al, 2001  (China; 1999-2000) | 93 / 98  H-B CCS. | **P. V.**  **(hOGG1-Ser/Ser)**  < once/w  ≥ once/w  **(hOGG1-Ser/Cys or Cys/Cys)**  < once/w  ≥ once/w | NR/NR  NR/NR | 1  1.10 (0.28-4.35)  1  1.47 (0.84-2.60) | 1. All cases had histologically confirmed OC. No information about subtypes.  2. Individually matched for age and sex.  3. Adjusted for age, sex, alcohol and tobacco use.  Note: the number of cases and controls with hOGG1-Ser/Ser genotype was 14 and 29 and with hOGG1-Ser/Cys or Cys/Cys genotype was 79 and 69, respectively. |
| Chen et al, 2004  (China;  1999-2000) | 100 / 100  H-B CCS. | **P. V.**  <once/day  ≥ once/day | NR/NR | 1  4.72 (1.95-11.46) | 1. All cases had histologically confirmed OC; only OSCC cases.  2. Individually matched for age, sex and residence.  3. Matched results adjusted for often getting angry when eating, often eat salted fish or meat and eat very rapidly. |
| Chitra et al, 2004  (India; 1999-2000) | 90 / 90  H-B CCS. | **Pickles**  Non-user  User | 16.7/33.3  83.3/66.7 | 1  2.5 (1.2-6.4) | 1. All cases had histologically confirmed OC; only OSCC cases.  2. Individually matched for age and sex.  3. Results were not adjusted. |
| Peng et al, 2005  (China;  2001-2002) | 237 / 237  H-B CCS. | **P. V.**  Frequency of use/w (continuous variable) | NR/NR | 2.28 (0.92-5.65) | 1. All cases were diagnosed by histology and X-ray or endoscopy; only OSCC cases.  2. Individually matched for age, sex and residence.  3. Adjusted for tea consumption, smoking, drinking clean water, salt consumption, fried food, moldy sweet potato, soy food, fruit, indrawn character, oesophageal cancer history of first-level relative. |
| Feng et al, 2008b  (China; 2001-2005) | 201 / 201  H-B CCS. | **P. V.**  < 3 times/w or duration < 5 ys  ≥ 3 times/w and duration ≥ 5 ys | 68.7/83.6  31.3/16.4 | 1  2.01 (1.15-3.54) | 1. All cases were diagnosed by endoscopy or histology. No information about subtypes.  2. Individually matched for age, sex, residence, nationality and diagnosed time.  3. Matched results adjusted for intake of meat and egg, tobacco use, preference to eat hot and hard diet, fierce mind stimulation and family history of oesophageal cancer. |
| Yang et al, 2005  (China; 2003-2004) | 185 / 185  CCS; H-B cases, P-B controls. | **P. V.**  < 1 meal/w  1-3 meal/w  ≥ 4 meal/w | 15.1/24.3  15.7/17.3  69.2/58.4 | 1  1.94 (0.77-4.89)  2.12 (1.00-4.49)  *P* for trend = 0.06 | 1. All cases had histologically confirmed OC. No information about subtypes.  2. Individually matched for age, sex.  3. Matched results adjusted for family history of OC, occupation, tobacco and alcohol use, some dietary items, including intake of tea, hot food, fruit and vegetables, and water supply. |
| Zhao et al, 2003  (China; period not reported) | 217 / 212  CCS. The origin of cases was not reported. Controls were healthy residents in a high risk village. | **P. V.**  Seldom  Often | 30.0/50.0  70.0/50.0 | 1  1.96 (1.26-3.05) | 1. No information about OC diagnosis methods or subtypes.  2. No matching was reported.  3. Adjusted for age, sex, education, drinking tea, and tobacco and alcohol use. |

Abbreviations: CCS, case-control study; OAC, oesophageal adenocarcinoma; OC, oesophageal cancer; OR, odds ratio; OSCC, oesophageal squamous cell carcinoma; hOGG1, human 8-hydroxyguanine DNA-glycosylase gene; NR, not reported; NS/NR, no statistically significant association, but OR was not reported; P., pickled; RR, relative risk; w, week; V., vegetable; y, year; 95% CI, 95% confidence interval.

^a^ If studies reported both crude and adjusted ORs (95% CIs), we only present the adjusted results.

***** These studies showed crude numbers but not ORs and 95% CIs; we calculated these statistics using simple logistic regression models and present them.
